# Supplementary material for: The impact of rehabilitation frequency on the risk of stroke in patients with rheumatoid arthritis
Source: PLoS One. 2020 Jan 13;15(1):e0227514. doi: 10.1371/journal.pone.0227514 (PMC6957159; doi:10.1371/journal.pone.0227514)
Supplement: Attachment — Submitted filename: Response to Reviewers.docx [file pone.0227514.s002.docx]

Journal Requirements:

1. The database we analyzed in this study was authorized by Taiwan National Health Insurance Administration, and could only be assessed in the Research Laboratory of our hospital. After statistical analyzing this database, the tables and figure in our manuscript are the only data we’re able to take out from the Research Laboratory. Therefore, I’m afraid that the original whole authorized database owned by our hospital cannot be provided under this circumstance.
2. Due to the anonymous nature of the national database, informed consent cannot be signed by each study subject. However, our study was still approved by the Institutional Review Board of Taichung Veterans General Hospital in Taiwan. (No. CE13152), and the ethics approval statement was included in “The Study Samples” section of “Material and Methods” in our manuscript.

Response to Reviewer #1:

Thanks for the detailed evaluation and critical comments on our study. We agree with your opinion that exercise is an important component to prevent stroke attack, especially in those with higher risk of stroke, such RA patients. We will try to reinforce this important point of view in our manuscript without overstating the presented findings. Thank you very much!

Materials and Methods:

1. We agree that the detailed content of exercise programs designed for RA patients should be mentioned in our manuscript. Since rehabilitation doctors and physical therapists in Taiwan always design exercise programs for patients according to ACSM's Guidelines for Exercise Testing and Prescription by American College of Sports Medicine, we added the following sentences in the section of “The Comorbidity Definitions, Groups Setting, and Study Design”: “As suggestions from American College of Sports Medicine, strengthening exercises are usually designed as 60-80% of 1 repetition maximum, 8-12 repetitions for 2-4 sets, 2-3 days per week, while cardiopulmonary endurance training emphasizes on exercises that maintaining the heart rate around 40-60% of heart rate reserve for an accumulation of 150 minutes per week.” We understand the concern that the rehabilitation sessions shown in our study seemed not enough to influence the risk of stroke. However, it’s just the times of rehabilitation visits recorded in the registry. During each visits, rehabilitation doctors and physical therapists in Taiwan serve as roles of reminding patients to maintain exercise trainings in their daily lives according to the suggestions from ACSM guidelines in addition to just doing exercise trainings.
2. As mentioned in our discussion, medications prescriptions in Taiwan were regulated by the rules set by Taiwan’s National Health Insurance Administration. Doctors cannot prescribe medications at their own will because National Health Insurance Administration, the single largest health insurance institution in Taiwan, will not pay for the drugs if rules are not followed. Certain criteria of disease severity should be fulfilled in order to be eligible for treatment with biologic agents, such as a Disease Activity Score (DAS 28) higher than 5.1 and treatment failure for six months using at least two kinds of DMARDs confirmed by photographic evidence and X-ray study. Therefore, we can use the medication types and dosage to classify the severity of diseases in studies utilizing the Taiwan NHI research database. Furthermore, the methods were also adopted in the past to stratify RA severity.[1]
3. We agree that the statistical adjustment information should be placed in the statistical analysis section in our manuscript. Thank you very much for reminding us!

Results:

1. In our study, we identified 663 RA patients with stroke at first, and then we tried to find the control subjects exactly matching for age, sex, and the time interval between RA and stroke diagnosis from 15,581 RA patients without stroke. However, only 642 patients could be matched in our database. After excluding patients whose time interval between RA certification and stroke index date less than 1 year, we obtained 591 RA patients with stroke and 582 RA patients without stroke in our study. There was no missing data. The numbers are not matched just because of we could not find enough patients exactly matching for age, sex and the time interval in our database.
2. Thanks for your reminding that we should be more specific here. The number of rehabilitation sessions here means the mean number of rehabilitation visits **per year** from the diagnosis of RA to the onset of stroke or index date. As we mentioned before, it’s just the times of rehabilitation visits recorded in the registry. During each visits, rehabilitation doctors and physical therapists in Taiwan serve as roles of reminding patients to maintain exercise trainings in their daily lives according to the suggestions from ACSM guidelines in addition to just doing exercise trainings.
3. As described in the title of table 2, the variables included in our final adjusted model are age, sex, hypertension, DM, hyperlipidemia, number of rehabilitation per year, and severity of RA. The model is like an equation “y=ax1+bx2+cx3+dx4+ex5+fx6+gx7+h”. While y is stroke, x1 to x7 are age, sex, hypertension, DM, hyperlipidemia, number of rehabilitation per year, and severity of RA, respectively. In the “Statistical Analysis” section, we described “…logistic regression analysis was conducted to determine the odds ratio of stroke based on age, sex, medical comorbidities, number of rehabilitation sessions per year, and RA disease severity with statistical adjustment for all the variables mentioned above”, which specified the variables in our final adjusted model.

Discussion:

1. We agree that the paragraph of discussion is long in its current form. However, the first paragraph summarizes the study findings, the second one discussed the method stratifying RA disease severity, the third one focused on why patients in the moderate disease severity had a higher risk of stroke, the fourth one showed the relationship between hyperlipidemia and stroke in RA patients in a past study, the fifth one discussed why exercise has protective effect in the moderate disease severity against stroke in our study, and the last one discussed the limitations of our study. After deliberate consideration, we decided to delete the four section of our discussion because the relationship between hyperlipidemia and stroke in RA patients is not the main focus of our study.
2. One of the major limitations of NHI research database is lack of lab data. As we mentioned in the discussion, many components of claims-based index for RA severity (CIRAS) such as rheumatoid factor, inflammatory markers, chemistry panels and platelet counts are not available in our registry. Instead, the advantage of our database is that the dosage of medications was available. Since medications prescriptions in Taiwan were regulated by the rules set by Taiwan’s National Health Insurance Administration, certain criteria of disease severity should be fulfilled in order to be eligible for treatment with biologic agents, such as a Disease Activity Score (DAS 28) higher than 5.1 and treatment failure for six months using at least two kinds of DMARDs confirmed by photographic evidence and X-ray study. Therefore, we can use the medication types and dosage to classify the severity of diseases in studies utilizing the Taiwan NHI research database. Furthermore, the methods were also adopted in the past to stratify RA severity.[1]
3. Thanks for noticing the interesting finding in our study, and we’ve adjusted the sentences of discussion here as: “By definition, biologic agents were only prescribed in the most severe group of RA patients in our study. Therefore, a possible reason is the anti-inflammatory effect of the biologic agents which would have reduced the likelihood of developing stroke in the most severe group in our study.…Hence, patients in the severe group, who were thought to have the highest risk of stroke, did not have a significantly higher risk compared to the mild group. On the contrary, without the potent anti-inflammatory effect of biologic agents, patients in the moderate group had a higher risk of developing stroke.”
4. Thanks for the valuable suggestion, and we’ve added the sentences below to better describe the detail of rehabilitation programs in Taiwan: “Since rehabilitation doctors and physical therapists in Taiwan always design exercise programs for patients according to ACSM's Guidelines for Exercise Testing and Prescription, the comprehensive rehabilitation programs in Taiwan regularly constitute of strengthening and aerobic parts of exercise training.”
5. We agree that more than 40 rehabilitation sessions per year cannot be considered as aggressive. Therefore, the adjectives “aggressive” or “intensive” have been avoided throughout our manuscript, and descriptions with more rehabilitation sessions are used instead.

References:

1. Tang KT, Chen YH, Lin CH, Chen DY (2016) Methotrexate is not associated with increased liver cirrhosis in a population-based cohort of rheumatoid arthritis patients with chronic hepatitis C. Sci Rep 6: 33104.

Response to Reviewer #2:

Design:

1. Thanks for the critical comment, and we also agree that risk factors of stroke such as age, sex, and medical comorbidities should all be matched between the study and control groups. However, in real world it’s essentially not possible to match these risk factors between stroke patients and non-stroke patients. In our study, we identified 663 RA patients with stroke at first, and then we tried to find the control subjects exactly matching for age, sex, and the time interval between RA and stroke diagnosis from 15,581 RA patients without stroke. However, only 642 patients could be matched in our database. If medical comorbidities including hypertension, diabetes and hyperlipidemia were to be matched in our study, the case number in the control group will be very few, rendering subsequent statistical analysis impossible and meaningless.
2. One of the major limitations of a retrospective study is the immortal time bias. However, our study avoided this bias by the nested case-control design matching the time interval between the study and control group. Another important issue is recall bias, which is also not possible to exist in our study because of the medical record registry-based in nature. About the appropriateness of the control group, we tried to match the potential confounding factors of stroke including age, sex, and the time interval between RA diagnosis and stroke. Other risk factors such as medical comorbidities were then statistically adjusted in further logistic regression models. We tried to ameliorate the disadvantage of a retrospective study by efforts mentioned above.
3. After our meticulous searching on the internet, we can still not find the reference for the cut-off times of rehabilitation sessions to be referred as intensive rehabilitation. As mentioned in our manuscript, 40 times of rehabilitation sessions per year roughly approximates to rehabilitation visits once per week after subtracting national holidays and vacations. Thank you for the critical comment, and we admit that the adjectives “intensive” or “aggressive” in our manuscript are not appropriate. Therefore, the adjectives “aggressive” or “intensive” have been avoided throughout our manuscript, and descriptions with more rehabilitation sessions are used instead.

Methods and results:

1. We’ve changed the term “hazard ratio” to “odds ratio”. Thank you very much for reminding us the important naming error.
2. Our logistic regression model is multivariate in nature. The model is like an equation “y=ax1+bx2+cx3+dx4+ex5+fx6+gx7+h”. While y is stroke, x1 to x7 are age, sex, hypertension, DM, hyperlipidemia, number of rehabilitation per year, and severity of RA, respectively. Indeed, after statistical adjustment for age, sex, medical comorbidities, number of rehabilitation sessions and severity of RA, the odds ratio of rehabilitation sessions for stroke attack did not achieve statistical significance. Only in the stratified analysis did we find significant odds ratio in the moderate severity group of RA patients.
3. The reason for us to do stratified analysis by RA severity is because we cannot find significant association between rehabilitation frequency and stroke incidence in the entire cohort we located. Since more physical activities brought by more frequent rehabilitation visits had been considered to be beneficial for stroke prevention, our study result using the entire cohort seems to be unreasonable. Therefore, stratified analysis was needed to clarify the results in the subgroups patients with RA. We also examined the interaction between number of rehabilitation sessions and severity of RA by adding their product terms into the logistic regression model (set at two-tailed p ≤0.05), which showed no significant interaction effects (P = 0.703).

Discussion:

1. We agree that some key discussions about the stratification of RA severity should be moved to the design section for better delineating the reason why we used the dosage of medications to categorize the severity. Therefore, the following paragraphs have been added in the design section: “The advantage of our database is that the dosage of medications was available. Since medications prescriptions in Taiwan were regulated by the rules set by Taiwan’s NHI Administration, certain criteria of disease severity should be fulfilled in order to be eligible for treatment with biologic agents. Therefore, we can use the medication types and dosage to classify the severity of diseases in studies utilizing Taiwan’s NHIRD.”
2. The third section of discussion focused on why patients in the moderate disease severity had a higher risk of stroke. Because the relationship between RA and stroke has been thoroughly studied in the past, comparing the stroke risk among different level of RA disease severity serves as a minor aim of our study. The major aim of this study is still the association between times of rehabilitation sessions and stroke incidence. Relevant changes have been made in our discussions.
3. We agree that different types, intensity and durations of exercise incorporated in the rehabilitation sessions may influence the incidence of stroke. However, the information of detailed exercise programs mentioned above cannot be obtained in the database we used. Nevertheless, rehabilitation doctors and physical therapists in Taiwan always design exercise programs for patients according to ACSM's Guidelines for Exercise Testing and Prescription, and the comprehensive rehabilitation programs in Taiwan regularly constitute of strengthening and aerobic parts of exercise training. We’ve added the above sentences in the discussion paragraph, and relevant details from American College of Sports Medicine were added in the design section.

Minor comments:

1. We admit that the adjectives “intensive” or “aggressive” in our manuscript are not appropriate. Therefore, the adjectives “aggressive” or “intensive” have been avoided throughout our manuscript, and descriptions with more rehabilitation sessions are used instead.
2. We will provide the STROBE checklist as a supplemental file. Thank you very much for your valuable suggestions.
