# Supplementary material for: The impact of rehabilitation frequency on the risk of stroke in patients with rheumatoid arthritis
Source: PLoS One. 2020 Jan 13;15(1):e0227514. doi: 10.1371/journal.pone.0227514 (PMC6957159; doi:10.1371/journal.pone.0227514)
Supplement: Attachment — Submitted filename: Response to Reviewer2.docx [file pone.0227514.s003.docx]

Response to Reviewer #2:

1. Thanks for the critical comment that it should be an important limitation in our study. We thus added the following sentences in our paragraph of study limitations in the discussion (Page 16 Line 284): “Third, while medical comorbidities including hypertension, diabetes and hyperlipidemia are important contributing factors of stroke, only statistical adjustments were done instead of matching. The reason lies in that if these medical comorbidities in our study were all matched, the case number in the control group will be very few, rendering subsequent statistical analysis impossible and meaningless. Therefore, only age, sex, and the time interval between RA and stroke diagnosis were statistically matched between the two study groups.”
2. We added “in the multivariate logistic regression analysis” in the table legends of Table 2 (Page 10 Line 189) and Table 3 (Page 11 Line 192). Thanks for your kindly reminding.
3. We revised our manuscript in the result section (Page 10 Line 178) as following: “…RA patients receiving more rehabilitation sessions did not have a significant reduction in risk of stroke attack (odds ratio=0.66, p=0.061). The results are shown in Table 2. Since physical activities brought by more frequent rehabilitation visits had been considered to be beneficial for stroke prevention, the result above seems to be unreasonable. Therefore, further stratified analysis on the effect of rehabilitation frequencies was done according to the disease severity.” Thanks for your valuable suggestion!
4. Thanks for your valuable advice, and we admit that it’s also one important limitation of our study. Therefore, the following sentences were added in our study limitations of discussion (Page 16 Line 299): “…Finally, the type, intensity, frequency and duration of exercise incorporated in the rehabilitation sessions may influence the incidence of stroke. However, the information of detailed exercise programs mentioned above cannot be obtained in the database we used. Therefore, further prospective study to address this gap is needed in the future.” Thanks again for your detailed review and critical comments on our study!
