# Supplementary material for: The impact of rehabilitation frequency on the risk of stroke in patients with rheumatoid arthritis
Source: PLoS One. 2020 Jan 13;15(1):e0227514. doi: 10.1371/journal.pone.0227514 (PMC6957159; doi:10.1371/journal.pone.0227514)
Supplement: Attachment — Submitted filename: Response to Reviewer3.docx [file pone.0227514.s004.docx]

Response to reviewers:

We’ve amended the term “multivariate” to “multivariable” in our manuscript. Thank you for the critical suggestion!
